# Financial Burden and Mental Health Among LGBTQIA+ Adolescent and Young Adult Cancer Survivors During the COVID-19 Pandemic

**DOI:** 10.3389/fonc.2022.832635

**Published:** 2022-06-16

**Authors:** Austin R. Waters, Sara Bybee, Echo L. Warner, Heydon K. Kaddas, Erin E. Kent, Anne C. Kirchhoff

**Affiliations:** ^1^ Cancer Control and Population Sciences Research Program, Huntsman Cancer Institute, Salt Lake City, UT, United States; ^2^ Department of Health Policy & Management, Gilling’s School of Global Public Health, University of North Carolina Chapel Hill, Chapel Hill, NC, United States; ^3^ College of Nursing, University of Utah, Salt Lake City, UT, United States; ^4^ Department of Pediatrics, University of Utah, Salt Lake City, UT, United States

**Keywords:** SGM, LGBTQIA^+^, AYA, financial hardship, financial toxicity, mental health

## Abstract

**Background:**

In the United States, the cost of cancer treatment can lead to severe financial burden for cancer survivors. The economic impacts of the COVID-19 pandemic compound cancer survivors’ financial challenges. Financial burden may be particularly challenging for lesbian, gay, bisexual, transgender, queer, intersex, asexual and other sexual and gender minority (LGBTQIA+) survivors. LGBTQIA+ survivors who are adolescent and young adults (AYA) may face elevated financial burden due to multiple, intersecting identities.

**Methods:**

An explanatory sequential mixed methods design was applied, beginning with a survey of AYA cancer survivors in the Mountain West region of the United States. Survey measures included demographics, COVID-19 impacts, the COmprehensive Score for financial Toxicity (COST), Perceived Stress Scale-4 (PSS-4), and PROMIS anxiety and depression scales. Two-way t-tests were used to analyze differences in outcomes between LGBTQIA+ and non-LGBTQIA+ AYAs. All LGBTQIA+ survey participants were invited to complete an interview, and those who agreed participated in descriptive interviews about financial burden due to cancer, COVID-19, and LGBTQIA+ identity. Interviews were audio recorded, transcribed, and analyzed using Dedoose.

**Results:**

Survey participants (N=325) were LGBTQIA+ (n=29, 8.9%), primarily female (n= 197, 60.6%), non-Hispanic White (n= 267, 82.2%), and received treatment during COVID-19 (n= 174, 54.0%). LGBTQIA+ interview participants (n=9, 100%) identified as a sexual minority and (n=2, 22.2%) identified as a gender minority. Most were non-Hispanic White (n=6, 66.7%) and had received treatment during COVID-19 (n=7, 77.8%). Statistical analyses revealed that LGBTQIA+ AYAs reported significantly worse COST scores than non-LGBTQIA+ AYAs (p=0.002). LGBTQIA+ AYAs also reported significantly higher PSS-4 (p=0.001), PROMIS anxiety (p=0.002) and depression scores (p<0.001) than non-LGBTQIA+ AYAs, reflecting worse mental health outcomes. High costs of cancer treatment and employment disruptions due to COVID-19 contributed to substantial financial stress, which exacerbated existing mental health challenges and introduced new ones.

**Conclusions:**

LGBTQIA+ AYA survivors reported substantial financial burden and psychological distress exacerbated by cancer, the COVID-19 pandemic, and LGBTQIA+ stigma. Given their multiple intersecting identities and potential for marginalization, LGBTQIA+ AYA survivors deserve prioritization in research to reduce financial burden and poor mental health.

## 1 Introduction

Rising costs of cancer care in the United States put a substantial proportion of cancer patients at risk of financial harm (1, 2). Financial burden due to cancer care is associated with poor economic, psychological, and physical health outcomes, and it may be worsened by the economic and psychological impact of the COVID-19 pandemic (3, 4). A variety of factors influence cancer patients’ likelihood of experiencing financial burden, including low socioeconomic status, younger age, minority race/ethnicity, social network wealth, employment disruptions, and health insurance access and quality (2, 5). While currently an unexplored area of research, adolescent and young adult (AYA) cancer patients, particularly those who are a part of the lesbian, bisexual, gay, transgender, queer, intersex, asexual, plus (LGBTQIA+) community may experience worse and unique financial burden in comparison to heterosexual, cisgender AYAs due to their multiple intersecting identities and experiences.

AYA cancer survivors are those who were diagnosed between the ages of 15 and 39, a developmentally dynamic time of life that positions them at greater risk of financial burden than older cancer survivors who often have more stable finances, careers, and health insurance coverage (6, 7). AYAs also have little to no experience navigating the healthcare system prior to their cancer diagnosis, potentially fostering financial burdens that those with experiential learning may know how to circumvent (e.g., knowing how to file insurance appeals, utilizing or having employment that allows for FMLA, or applying for financial aid) (6, 8).

LGBTQIA+ populations of all ages experience disparities within and outside of the cancer context that may influence LGBTQIA+ AYA survivors’ financial burden (9, 10). Furthermore, identity development and the process of coming out for LGBTQIA+ individuals typically occurs during adolescence and young adulthood (11, 12). LGBTQIA+ identity development during the cancer experience can further complicate the already intersecting identities of this population and may lead to additional burden (13). Among LGBTQIA+ populations, some sub-groups of the community have lower incomes and experience workplace discrimination more often than their non-LGBTQIA+ peers (14, 15). Due to intertwined structural and interpersonal factors, LGBTQIA+ individuals are more likely to struggle with mental health issues, exhibit negative coping behaviors such as binge drinking, and are more likely to commit suicide than cisgender, heterosexual individuals (16, 17) Within the cancer context, LGBTQIA+ populations experience a disproportionate cancer burden, provider-based discrimination, unwelcoming cisheternomative clinic spaces, worse mental health, and cancer morbidity (18–20).

The aim of this study was to first assess differences in financial burden and mental health outcomes between LGBTQIA+ and non-LGBTQIA+ AYA survivors during the COVID-19 pandemic. Second, we aimed to describe how the COVID-19 pandemic, cancer treatment, LGBTQIA+ identity and related stigma impacted LGBTQIA+ AYA survivors’ financial burden and mental health. Our findings underscore the importance of considering intersecting identities and the historical and structural forces that influence LGBTQIA+ AYA survivors’ financial burden. These findings serve as a first look at an understudied population and have the potential to inform future research and equity-based interventions aimed at mitigating financial burden.

## 2 Materials and Methods

To describe LGBTQIA+ AYA survivors’ financial burden during the COVID-19 pandemic, we deployed a sequential explanatory mixed methods study design (21). First, we surveyed AYA cancer survivors of all sexual orientations and gender identities who received AYA patient navigation services in the Mountain West region of the United States. Survey findings documented differential financial burden among LGBTQIA+, which led us to conduct one-on-one semi-structured video interviews with a subset of AYA survivors who identified as LGBTQIA+.

### 2.1 Participants and Data Collection

Eligible survey participants were 18 years or older at time of survey, diagnosed with cancer between the ages of 15 and 39 years, and received services through the Huntsman-Intermountain Adolescent and Young Adult (HIAYA) Cancer Care Program in Utah, which serves AYAs with cancer in the Intermountain West region of the United States. All survivors who had received services through HIAYA were emailed a link to the one-time survey in October 2020. A total of 675 survivors were eligible and contacted *via* email. Follow up occurred between October 2020 to January 2021 *via* email, mail, and text messages, resulting in 341 participants (response rate of 50.5%). Our survey analyses are restricted to respondents who completed the sexual orientation and gender identity questions (N=325).

Participants who were eligible for the LGBTQIA+ interviews took part in the larger AYA survey and self-identified as LGBTQIA+. A total of 29 (8.9%) participants from the larger survey sample (N=325) self-identified as having a sexual orientation or gender identity other than heterosexual, cisgender, and binary and were therefore categorized as LGBTQIA+. Of these, there were 25 respondents who agreed to be re-contacted for future research. These potential participants were emailed an invitation to participate in an individual semi-structured video interview between August and November 2021 and received follow-up emails and text messages inviting them to take part in an interview about their financial experiences. Potential participants who agreed to partake in the interview completed the informed consent process and engaged in an interview *via* videoconferencing software. All interviews were conducted by ARW, a male doctoral student in public health with four years of experience in AYA cancer research. Nine participants agreed to be interviewed (participation rate of 36%). Six participants declined to participate (often citing their willingness to participate in a survey but not an interview), three were lost to follow up, and seven were unable to be contacted. Participants received one $20 gift card for participating in the survey or two $20 gift cards for participating in both the survey and interview as a thank you for their time. All study procedures were approved by the University of Utah Institutional Review Board (IRB#00091443).

### 2.2 Survey Design

Survey questions included sociodemographics, cancer diagnosis, mental health, and the COVID-19 pandemic. The HIAYA research team, which includes health services researchers, clinicians, and research staff with expertise in AYA cancer and LGBTQIA+ research, designed the survey for a larger study to document the financial experiences and healthcare utilization of AYA cancer survivors during the COVID-19 pandemic. Herein we report on selected items relevant to LGBTQIA+ survivors’ financial and mental health experiences during COVID-19.

### 2.3 Survey Measures

Outcome measures from the survey included: COmprehensive Score for financial Toxicity (COST), Perceived Stress Scale – 4 (PSS-4), Patient-Reported Outcomes Measurement Information System (PROMIS) short form measures for anxiety, and a custom short form PROMIS measure for depression. This custom short form was created with the cancer population as a control sample and included 7 items in the cancer depression bank and was scored through the PROMIS Assessment Center (22). COST is a measure of perceived financial stress due to cancer treatment. COST scores range from 0-44 with lower scores indicating greater financial toxicity (23, 24). PSS-4 is a measure of perceived stress with scores ranging from 0-16 with higher scores indicating greater stress (25, 26). The PROMIS anxiety short form is a measure of perceived anxiety with scores ranging from 37.1-83.1 with higher scores indicating worse anxiety (27). The custom PROMIS depression short form is a measure of perceived depression with scores ranging from 38.3-81.5 with higher scores indicating worse symptoms of depression.

The study team reviewed medical records to supplement missing demographic (e.g., gender, race, and ethnicity) information. We combined two survey variables (employment status and changes in employment during the COVID-19 pandemic) to operationalize change in employment status (still employed, decrease in hours/job loss, and increase in hours). Individuals who wrote in responses for their employment status and change in employment status were manually categorized. Treatment status was dichotomized (on treatment/off treatment) based on type of treatment that they were receiving at time of survey (intravenous chemotherapy, oral chemotherapy/pills, surgery, radiation, hormone therapy, immunotherapy, and/or other treatment). Individuals who wrote in responses for their treatment status were manually categorized, and those who responded they were not currently undergoing any of these treatment types were classified as being off treatment. Age at diagnosis was calculated from date of birth and date of first cancer diagnosis. Age at diagnosis was dichotomized (18-26 years/27-39 years), due to changes in insurance coverage that occur at this age in the United States (28). Education was collapsed to three categories (college graduate or higher, some college, high school education or less). Race and ethnicity were collapsed into a single variable (non-Hispanic White, Hispanic, non-Hispanic other). Information on sexual orientation and gender identity were dichotomized (cisgender heterosexual/LGBTQIA+).

### 2.4 Survey Data Analysis

Chi-square or Fisher’s Exact tests were applied to examine sociodemographic differences between individuals identifying as LGBTQIA+ and individuals identifying as cisgender, heterosexual. For each outcome measure (COST, PSS-4, PROMIS anxiety and depression), two-way t-tests were used to examine differences in the mean between individuals who identified as LGBTQIA+ and individuals identifying as cisgender, heterosexual. Significance was set at p<0.05. All analyses were done in STATA 17 (College Station, TX: StataCorp LLC).

### 2.5 Interview Guide Design

Upon discovering higher financial toxicity among LGBTQIA+ AYAs and the inability to explore driving forces of financial burden in the survey data, our interview guide was developed to disentangle drivers of financial hardship and explore unique experiences faced by LGBTQIA+ AYAs during the COVID-19 pandemic. The interview guide focused on how LGBTQIA+ AYA survivors’ cancer, the COVID-19 pandemic, and their LGBTQIA+ identity impacted their financial experiences. The interview guide was modeled to encompass three domains of financial hardship: 1) Material – out of pocket expenses, employment issues, and ability to meet financial needs; 2) Psychological – stress experienced due to costs and lost income; and 3) Behavioral – coping behaviors engaged in as a response to financial hardship including changes in health service utilization and adherence, as well as changes to non-healthcare spending (29, 30). In this analysis the three domains of financial hardship used in the interviews and the COmprehensive Score for financial Toxicity (COST) was used in the survey. Both financial hardship and financial toxicity were used to assess overall financial burden of LGBTQIA+ AYA survivors.

### 2.6 Qualitative Data Analysis

Interviews were audio recorded, transcribed, and quality checked for accuracy of transcription. They were then de-identified and imported into Dedoose qualitative analysis software. Interpretive descriptive methods of analysis were applied to provide an in-depth account of the financial burden experienced by AYA LGBTQIA+ survivors. Interpretive description is a qualitative technique that acknowledges the constructed nature of experiences of phenomenon but also allows for shared realities (31, 32). This analytical approach is particularly well suited for describing LGBTQIA+ AYA survivors’ experiences with cancer, COVID-19, and their LGBTQIA+ identity because of the focus on a strategic synthesis of new understanding and clinical applications (31, 32). As the transcripts were coded, emergent concepts were labeled and emergent codes were sorted into the three financial hardship domains (material financial hardship, behavioral financial hardship, and psychological financial hardship) and an additional domain called mental health challenges. The research team first read through all interview transcripts to gain familiarity with the content and created analytic memos (33). ARW then coded 33% of the interviews to create the initial coding matrix. ARW and SB then coded an additional 33% of the interviews and refined the coding matrix *via* coder consensus. Coder consensus is an activity wherein all coders agree on the labeling of each code within a sub-set of the transcripts to ensure the coding structure is reliably and consistently applied (34). A finalized coding matrix was developed *via* coder consensus (ARW, SB, and ELW) and then used to code all transcripts. To maximize reflexivity, interviews and qualitative analyses were conducted iteratively.

Qualitative analyses were performed by ARW, SB, and ELW in Dedoose; interpretation of the data occurred through iterative weekly author discussions to gain consensus and consistency of the reported findings. The research team approached the analysis and interpretation of the codes from a variety of lenses and identities including “insider” and “outsider” perspectives (i.e., LGBTQIA+ as well as cisgender, binary, heterosexual researchers).

### 2.7 Data Integration

**Figure 1:**
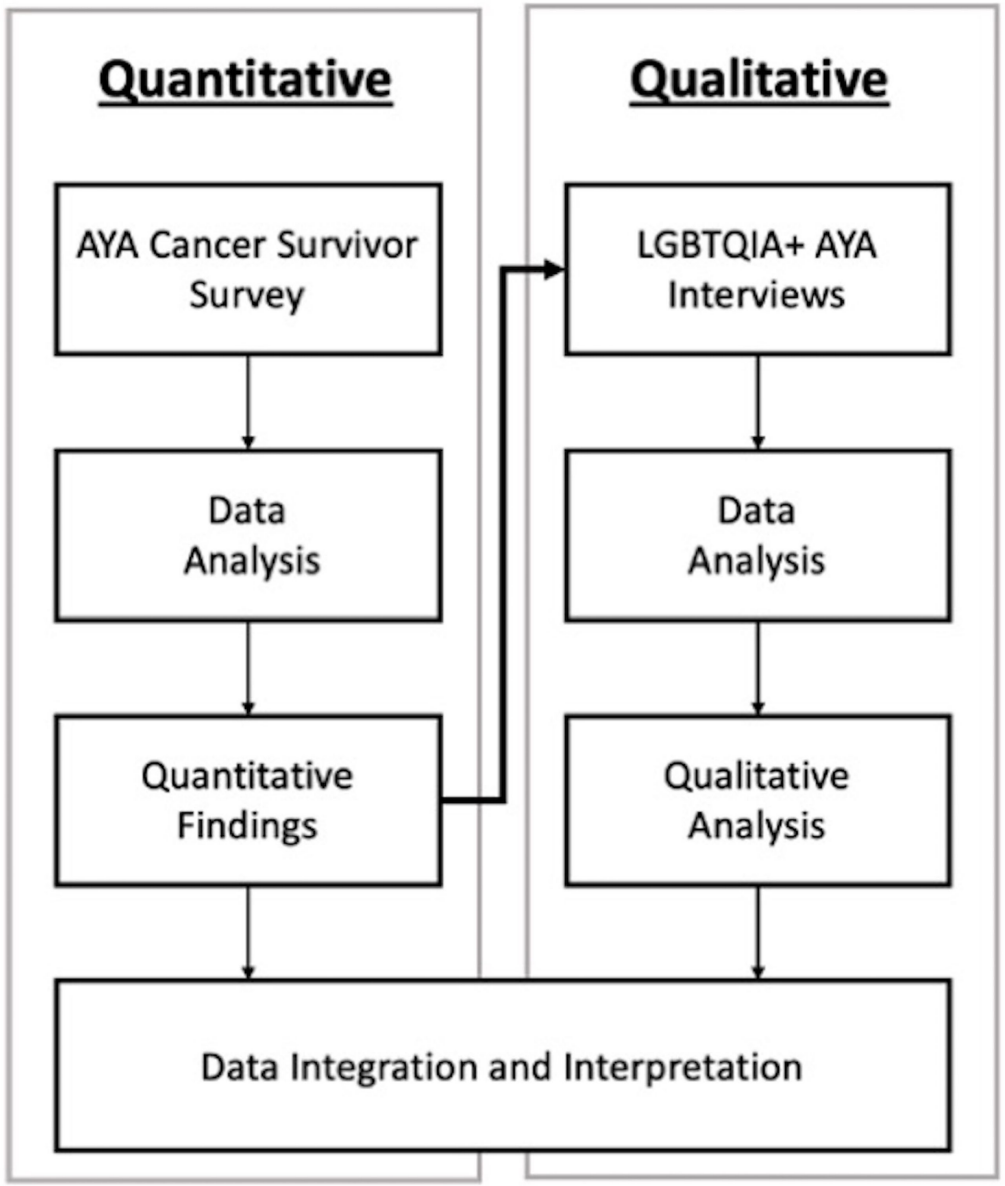
Explanatory Sequential Mixed Methods Study Design Diagram.

**Figure 2:**
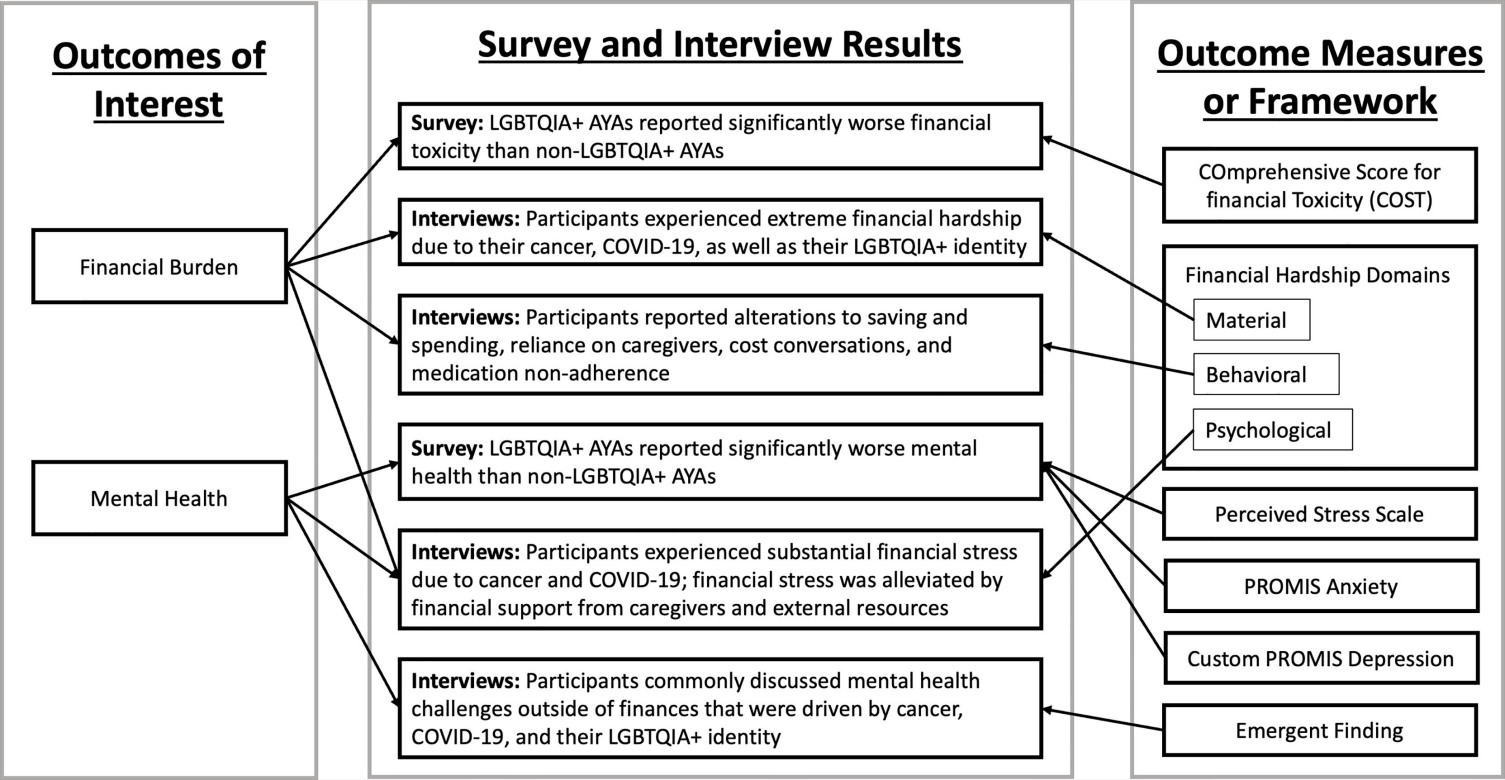
Integration of Survey and Interview Results and Corresponding Outcome Measures or Framework.

Data integration occurred at all stages of the study. In conceptualization, an explanatory sequential mixed methods design was chosen to first identify differences in financial hardship among AYA cancer survivors by demographic factors (e.g., LGBTQIA+ identity) and then to explore drivers of those differences using individual interviews (**Figure 1**). Integration *via* connecting also occurred through the sampling frame, meaning that interviewees were a subset of survey participants; thus, interview participants’ feedback is connected to the survey results because these participants took part in both the survey (quantitative) and interview (qualitative) portions of the study (35). Lastly results were integrated using a weaving approach in which survey and interview findings are reported in the results by concept rather than analytical method (21, 35). Integration also occurred in the creation of **Figure 2** that visualizes how survey and interview data are presented *via* the weaving approach to data integration. Each finding in **Figure 2** was also linked back to our outcomes of interest and the outcome measure or framework that was used to capture each outcomes of interest in both the survey and interview findings.

## 3 Results

In **Table 1**, survey participants (N=325) were primarily female (60.6%), non-Hispanic White (82.2%), and received cancer treatment during COVID-19 (54.0%). Nearly half were college graduates or higher (46.0%) and 21.9% reported a decrease in hours or job loss during the COVID-19 pandemic. Differences between LGBTQIA+ and cisgender heterosexual AYA survey respondents included more LGBTQIA+ respondents identifying as female (p-value=0.001), reporting less education (p-value=0.003), and a higher proportion reporting decrease in hours or job loss (p-value=0.001). LGBTQIA+ interview participants (N=9) all identified as sexual minority (100%), while two of the participants also identified as a gender non-binary (22.2%). Interview participants were mostly non-Hispanic White (66.7%) and had received cancer treatment during COVID-19 (77.8%). Most interview participants were college graduates or higher (55.6%) and most reported a decrease in hours or job loss during the COVID-19 pandemic (66.7%). Survey and interview findings are reported by the two main outcomes of interest – financial burden and mental health – visualized in **Figure 2**.

**Table 1:** Characteristics of Quantitative Survey and Qualitative Interview Participants and Differences by LGBTQIA+ Status among Survey Participants (N=325).

Sociodemographic Factors	Surveys							Interviews	
	Total (N=325)		LGBTQIA+ (N=29)		Cisgender, Heterosexual (N=296)		p-value	LGBTQIA+(N=9)	
	N	%	N	%	N	%		N	%
Age at Diagnosis									
18-25 years	164	50.5	17	58.6	147	49.7	0.20	6	66.7
26-39 years	161	49.5	12	41.4	149	50.3		3	33.3
Gender									
Non-binary	2	0.6	2	6.9	–	–	0.001	2	22.2
Female	197	60.6	21	72.4	176	59.5		6	66.7
Male	126	38.8	6	20.7	120	40.5		1	11.1
Ethnicity and Race									
Non-Hispanic White	267	82.2	22	75.9	245	82.8	0.07	6	66.7
Hispanic	30	9.2	6	20.7	24	8.1		2	22.2
Non-Hispanic other	28	8.6	1	3.5	27	9.1		1	11.1
Education^a^									
College grad or higher	149	46.0	5	17.9	144	48.7	0.003	5	55.6
Some college	139	42.9	17	60.7	122	41.2		4	44.4
High school education or less	36	11.1	6	21.4	30	10.1		–	–
Employment Status Changes During Pandemic^b^									
No change	176	56.9	7	26.9	169	59.3	0.001	1	11.1
Decrease in hours/job loss	68	21.9	13	50.0	55	19.3		6	66.7
Increase in hours	67	21.5	6	23.1	61	21.4		2	22.2
Received Cancer Treatment During Pandemic^c^									
Yes	174	54.0	17	58.6	157	53.6	0.60	7	77.8
No	148	46.0	12	41.4	136	46.4		2	22.2

a Missing N=1.

b Missing N=14.

c Missing N=3.

The N=9 interview participants were a sub-set of the N=29 LGBTQIA+ survey participants.

p-values were calculated using Chi-squared or Fisher’s exact tests.

### 3.1 Financial Burden: Toxicity and Hardship

**Figure 3:**
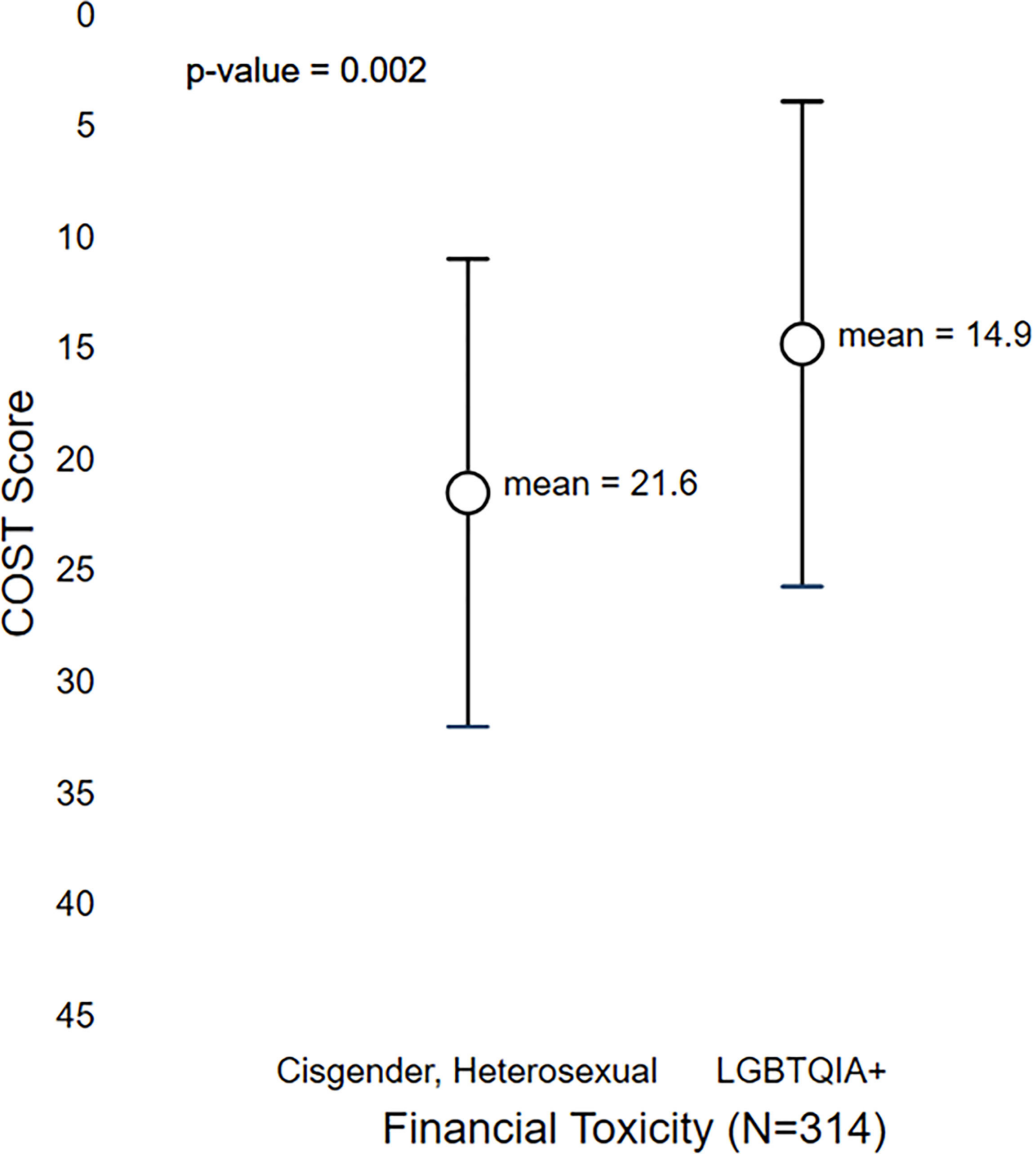
Differences in COST Scores Between Cisgender, Heterosexual and LGBTQIA+ AYA Cancer Survivors.

In the survey, LGBTQIA+ AYAs reported a mean COST score of 14.9 (SD=10.9) while cisgender, heterosexual AYAs reported a mean COST score of 21.6 (SD=10.5). LGBTQIA+ AYAs COST scores were significantly lower, indicating worse financial burden than non-LGBTQIA+ AYAs (p=0.002; **Figure 3**). These survey findings can be grounded by the interview findings which are described under the three domains of financial hardship (29). The interview findings provide context for how financial hardship manifested in LGBTQIA+ AYAs due to their identity, cancer, and the COVID-19 pandemic. Additional illustrative quotes are in **Table 2**.

**Table 2:** Material and Behavioral Financial Hardship – Sub-categories and Illustrative Quotes.

Sub-categories	Illustrative Quotes
Material Domain of Financial Hardship	“I was not fired, but I was under a pay freeze and asked to take on continuously more and more work, while I was still doing chemotherapy treatment” - Non-binary participant 18-25 years of age “Yeah, but – yeah. No, it was definitely still a challenge because when I was going through treatment, throughout all of treatment, and then for the first couple months afterward, I was making almost half of what I do now.” - Non-binary participant 18-25 years of age “It came to a time where I had to have three [procedure/scans] in less than three months, so just on that, it was $2,100.00 out of my pocket that I needed to pay that. And then, on top of that, there was a lot of copays. Some of them were not much. Some of them were higher, but dime by dime you make a million.” - Female participant 18-25 years of age “Being in a more queer accepting job that also has recognized that I have talent and have capability has been a really big boon for me. And I would not be in the same position both financially and out to my work community if I had stayed in [my old job].” - Non-binary participant 18-25 years of age “Yeah [I’m not out], it’s usually just fear of rejection, because people just kind of treat you differently, or weirdly, or like, “Okay, that’s weird.” - Non-binary participant 26-39 years of age “I haven’t been without a job because I was gay. I have turned down jobs – good paying jobs – because of bosses [who were weird about my LGBTQIA+ identity]” - Male participant 18-25 years of age
Behavioral Domain of Financial Hardship	“But you know, that’s also been stressful because I also wanna save, I wanna buy a house, and then it’s just too much bills on top of too much bills” - Male participant 18-25 years of age “Life pre-diagnosis and pre-COVID, I mean, I had a little bit more of that flexibility of being able to spend money on fun things for myself. Soon as cancer hit, that entirely mentality had to go away. It was pretty much like, if this is not an essential need, you don’t need it. So, this is not something you get right now. Or if you really want something like that, then maybe you can ask your nice friends to take care of things for you because there’s a lot of people who really wanna know how they can help right now.” - Non-binary participant 26-39 years of age “My husband was still taking care of other bills that were much major and much need of a more attention to also because my health is important, but we still need a roof over our head.” - Male participant 18-25 years of age “I’m just kind of existing. I was a full-time cancer patient for my treatment, and right now, I’m just living at home. My spouse has a full-time job with good health insurance, so that’s kind of what I’m living off of right now.” - Female participant 26-39 years of age

#### 3.1.1 Material Domain of Financial Hardship

Interview participants faced substantial financial hardship influenced by the high costs of cancer care, the economic impacts of the COVID-19 pandemic, and the impact of their LGBTQIA+ identity on their economic mobility. Many participants reported being laid off or having their hours/pay reduced due to the COVID-19 pandemic, which was further complicated by their increased susceptibility to COVID-19 due to their cancer status. One female survivor (26-39 years of age) shared *“It was very difficult because I lost two of my jobs that were giving me that income.”* Another non-binary participant (18-25 years of age) stated *“I was barely able to afford rent at the time, rent, and cancer treatment, and all of that [COVID-19] at the same time.”* Many participants worked customer facing jobs prior to the pandemic that led some participants to avoid working out of fear of being infected with COVID-19. However, some participants reported situations in which they received employer accommodations after being diagnosed with their cancer to protect them from COVID-19 infection, such as being moved to a less customer facing role. One female participant (18-25 years of age) shared *“It’s just hard. Because I don’t want to be exposed to anybody or anything like that. I feel like I couldn’t work any jobs that involve interacting with other people.”* Overall, participants reported that together COVID-19 and cancer drastically reduced their income and ability to make ends meet financially.

Furthermore, some participants reported their LGBTQIA+ identity impacted their material conditions in the form of employment discrimination, which was dependent on their outness. Few participants reported overt discrimination the workplace; however, many participants who were out reported taking lower paying jobs or leaving jobs to find more queer-accepting employment environments, which often manifested in lower paying, customer facing employment. Although one participant reported being called slurs related to their sexual orientation in the employment setting, more commonly participants reported employers “*being weird*” about their identity. One male (18-25 years of age) participant shared *“I had a customer here and there that were just*, *“Oh, you fucking [LGBTQIA+ slur],” you know.”* Another participant (female aged 18-25 years) shared their perception on employers being weird by stating *“One of the ladies who was in there, her eyebrows kind of raised. I’m not gonna say they didn’t hire me for that [being LGBTQIA+]. I honestly think it was my schedule because I didn’t really know my schedule if I was gonna be sick from the medicine, you know, all of that, but I know they were kind of weird about it.”* This weirdness was identified by participants as a factor that influenced not being hired for a job or choosing not to take a job due to their identity. However, discrimination was not reported by participants as the main cause of not receiving an offer of employment. Loss of employment or taking lower paying jobs among out participants further exasperated financial hardship caused by high out-of-pocket costs and instability of income due to COVID-19.

#### 3.1.2 Behavioral Domain of Financial Hardship

In response to financial hardship experienced, participants reported a variety of behavioral responses including: alterations to saving and spending habits; a reliance on caregivers and other external mechanisms for financial support; having cost conversations with clinicians and supportive healthcare staff; and rationing of prescription medications. The most commonly reported behavioral response to financial hardship was alterations to spending and saving habits. This reduction in spending ranged from small alterations (e.g., not eating take-out as frequently) to large life-altering spending changes (e.g., moving in with parents when unable to pay rent). A female participant aged 26-39 years of age shared *“And before I got diagnosed with cancer, I was actually living like, in [the city] on my own and then I had to – I couldn’t afford anything, so I had to move out and I had to move back in with my parents.”* In an extreme case, one participant reported losing their job and becoming homeless. Extreme outcomes, such as homelessness, were driven by job loss resulting from COVID-19 combined with familial non-acceptance of LGBTQIA+ identity. It was common for participants to rely financially on caregivers and crowdfunding platforms; however, when participants were not accepted by their families due to their LGBTQIA+ status, they lost the corresponding financial support. One participant discussed not coming out because of the potential loss of financial support during their cancer treatment and because they already felt marginalized as a person of color.

When asked about their experience and or willingness to discuss treatment costs with providers, most participants indicated they had spoken with a member of their care team or that they were willing to consider having a cost conversation with providers. Few participants reported in-depth conversations with medical providers about costs. Participants were frequently referred to social workers, patient navigators, or hospital financial aid services. Some participants found the resources and aid were extremely helpful while others were frustrated because they did not meet eligibility requirements. In particular, one participant was unable to receive aid because they did not have US citizenship. When cancer costs were unmanageable, participants reported medication non-adherence including skipping doses or delaying filling prescriptions for weeks to months until they could afford the co-pay. One participant (non-binary, aged 26-39 years) shared their experience with skipping unaffordable prescription medication by stating *“Yeah. Like, I mean, a lot of my prescriptions are really expensive. And for instance, one of my prescriptions is $1,500 a month, and that’s just one of them. I have several that are, like, $1,000. And, my insurance wouldn’t pay for it a couple of months ago, and, I just went without it for a month because I couldn’t afford to buy it.”* In addition to medication non-adherence, some participants partook in drastic behaviors to cope with situations that arose because of their financial hardship. For example, one participant stopped cancer treatment after losing their health insurance, because they were laid off due to COVID-19: *“I had to stop [treatment] because I lost my insurance [when I lost my job],”* shared a female participant 26-39 years of age. Another reported intentionally infecting themselves, *via* intravenous drug use, with an infectious disease so they would be eligible to receive free treatment for the infectious disease, which they perceived could also be used as an off-label treatment for their cancer that they could not afford otherwise.

### 3.2 Psychological Financial Hardship and Mental Health

**Figure 4:**
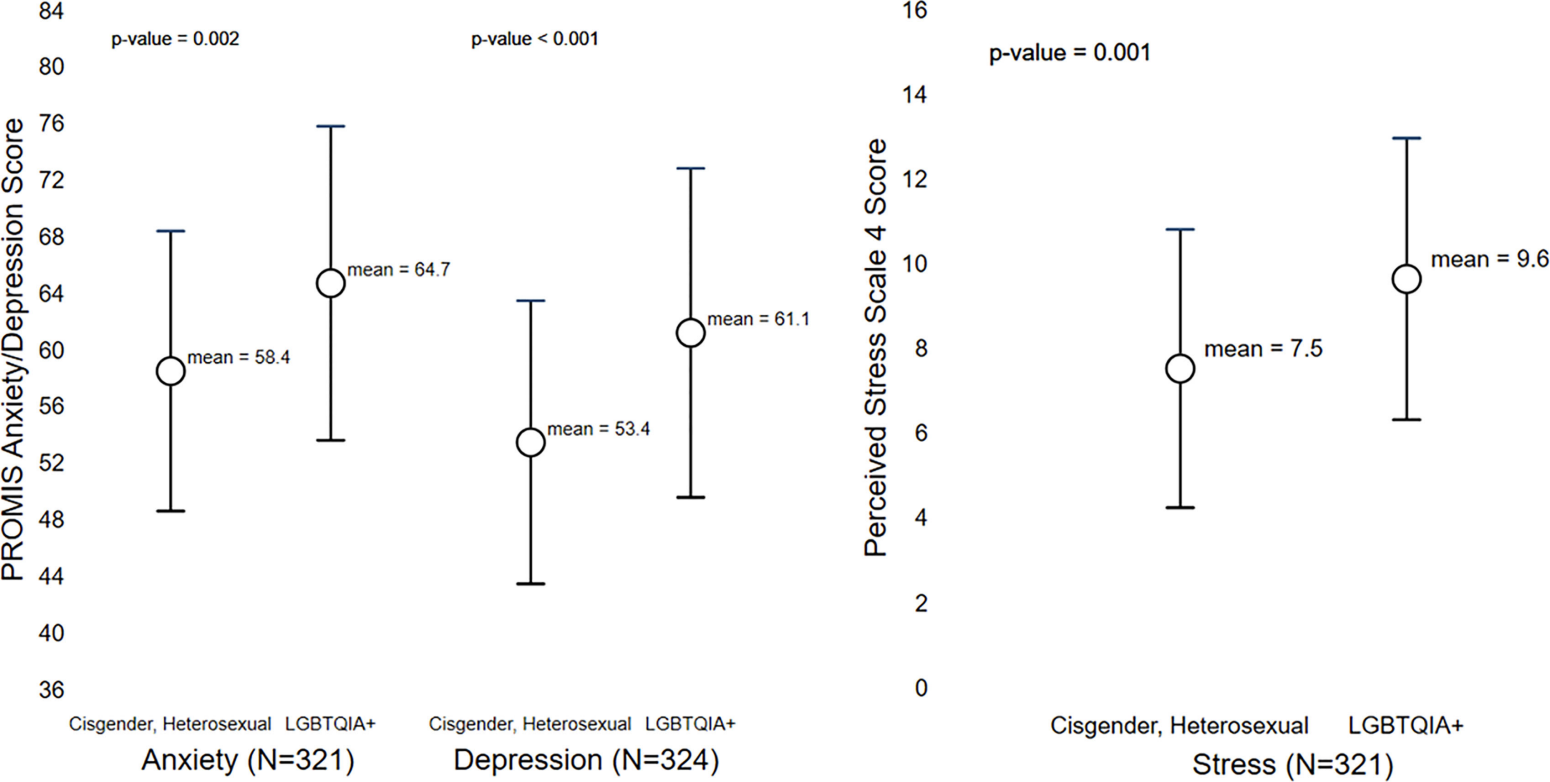
Differences in Mental Health Outcomes Between Cisgender, Heterosexual and LGBTQIA+ AYA Cancer Survivors.

LGBTQIA+ AYAs reported significantly worse stress (mean=9.6 [SD=33] *vs*. 7.5 [3.3]; p=0.001), anxiety (64.7 [11.1] *vs*. 58.4 [9.9]; p=0.002), and depression (61.1 [11.6] *vs*. 53.4 [10.0]; p<0.001) scores in comparison to cisgender, heterosexual AYAs (**Figure 4**). Interviewees explained that financial hardship resulted in substantial financial stress which was exacerbated by existing mental health challenges experienced by LGBTQIA+ AYAs. Existing mental health challenges related to social support, acceptance, and LGBTQIA+ identity emerged as integral to the psychological impact of financial hardship but persisted as a distinctly different topic explored below. Additional, illustrative quotes can be found in **Table 3**.

**Table 3:** Psychological Financial Hardship and Mental Health Challenges – Sub-categories and Illustrative Quotes.

Sub-categories	Illustrative Quotes
Psychological Domain of Financial Hardship	“I had nothing left of my life [after coming out and going through treatment] … And it was lonely. And it was hard. And it was scary. And it was painful” - Female participant 26-39 years of age “I think it impacted it [the COVID-19 pandemic] just in terms of thinking. Like, okay, well, what if I do get really sick and I’m not able to work?” – Non-binary participant 26-39 years of age “But yeah, I guess looking to the future, too, my cancer can come back within the next two years is kind of how it behaves, so I’m trying to just think ahead and be smart about financial decisions to be prepared next time if it ever comes back, which I hope it doesn’t.” - Female participant 26-39 years of age “I don’t feel [stressed] now other than, looking forward at like, what scans will I need? Like, if I get cancer again, how will I handle that financially?” – Non-binary participant 26-39 years of age
Mental Health Challenges	“it’s pretty much stressful because you never know if any little thing could be cancer, or any little thing could not be cancer” - Male participant 18-25 years of age “I struggled a lot with mental illness in my early 20s and I think I kind of, that was the priority. It’s like Maslow’s needs, you know, that staying alive was the priority” – Non-binary participant 26-39 years of age “So many times this year, I got put into a box that I didn’t belong in. All these boxes, everybody kept shoving me in. And I was, “Don’t put me in your box.” So, that’s my new thing. But they keep putting me in a box. Not fair” - Female participant 26-39 years of age “Also the fear of overt discrimination or anything like that, of more like, subconscious discrimination. So, you know, even smaller things, just like, to this day I’m still terrified” - Female participant 18-26 years of age

#### 3.2.1 Psychological Domain of Financial Hardship

The stress participants felt in response to the financial hardship was substantial. Nearly all participants reported feeling highly stressed due to the overlapping of COVID-19 and their cancer. A female participant (26-39 years of age) shared,*”It was like – it was 100 percent [stressful]. It’s on a scale of like 1 to 10, it would probably be a 12 only because like, in that timeframe, it was the worst. It was COVID and then my cancer diagnosis, those were probably the most stressful times of my life.”* Some participants talked about their cancer and COVID-19 experiences as the most stressful of their lives. Participants who reported being the most stressed were those receiving treatment or still paying bills from treatment during the pandemic. Participants commonly mentioned that resources provided by the cancer center, financial support from caregivers, expanded governmental unemployment/stimulus checks, and crowdfunding helped alleviate stress. However, some participants continued to feel a sense of despair about their financial situation when they felt they had run out of options for support. This feeling was shared by a female participant (aged 26-39 years): *“I’ve wrung out my resources. There is nothing left. I am an AYA girl. I can call every number, every email. I can fill out every application in that whole place, especially the [Cancer Center] resource booklet that they got for you. Oh, man, that resource book got me through. I am out of [financial] choices.”* Feelings of despair, fear, and loneliness caused by financial hardship were not uncommon among participants but were substantially elevated among those with existing mental health challenges.

#### 3.2.2 Mental Health Challenges

Unprompted by the interview guide, many participants discussed previous challenges with mental health that occurred before their cancer diagnosis and COVID-19 to contextualize the toll that their cancer and COVID-19 had taken on their mental health. One non-binary participant aged 26-39 years shared their mental health challenges prior to diving into how the questions being asked fit in their life stating, *“I actually had some mental health problems, and that’s another thing I didn’t talk about at all actually.”* Participants often discussed prior mental health challenges, such as being institutionalized or traumatic loss of family members, as a starting point for the impact of their financial stress. Participants felt it important to first explain their mental health prior to the COVID-19 pandemic and cancer diagnosis to fully describe how the financial stresses layered on top of their existing challenges.

Most participants who were off treatment reported anxiety surrounding recurrence. Many participants were fearful of being infected with COVID-19 due to their increased susceptibility to severe infections as a cancer patient. [quote] Furthermore, discussion revolving around LGBTQIA+ identity and mental health was common. Some participants reported not being accepted by family due to LGBTQIA+ identity and this nonacceptance having a severely negative impact on their mental health. One female participant, aged 26-39, reported doing drugs to cope with the mental toll of identity non-acceptance during cancer stating, *“After all of that loss [due to non-acceptance], I kind of started self-medicating ‘cause why not?”* Other participants who were not out felt fear of discrimination or loss of relationships and support if their family or employers (i.e., sources of financial support) learned of their LGBTQIA+ identity. In one case, a non-binary participant aged 26-39 was currently experiencing suicidal ideation at the time of interview due to conflict with a familial caregiver surrounding their LGBTQIA+ identity and dependence on that caregiver for financial and other support, stating: *“Yeah. Well, I do think so [that my prior mental health challenges were due to my LGBTQIA+ identity]. I’m gonna cry a little because, I mean, you don’t feel good when you can’t be yourself and when you feel like you have to pretend … I mean, that is the one thing that just makes me not want to be alive.”*

## 4 Discussion

Our findings suggest that LGBTQIA+ AYA survivors face substantial financial burden and mental health challenges that were enhanced by the ongoing economic and psychological uncertainty from COVID-19. Financial burden was often driven by intertwined factors including their cancer, COVID-19, and stigma surrounding their LGBTQIA+ identity. While financial burden among LGBTQIA+ AYA survivors has not been explored previously, AYA cancer survivors of all sexual orientations and gender identities are at an elevated risk of financial hardship compared to older adults due to high cancer related out-of-pocket costs (36, 37). Our findings fit into the existing literature by highlighting an unexplored demographic group of cancer survivors at risk for severe financial burden. In general, the COVID-19 pandemic has impacted both LGBTQIA+ and young adults’ financial burden and mental health more severely than non-LGBTQIA+ and older individuals (38, 39), which is consistent with the findings of our study.

Due to the largely unexplored nature of financial hardship in the LGBTQIA+ AYA survivor population, we first identified theoretical and conceptual underpinnings within and outside of the cancer context to begin to root our findings into the literature. The Sexual and Gender Minority (SGM) Health Disparities Research Framework provides a theoretical basis for interpreting our finding of disproportionate adverse outcomes experienced by LGBTQIA+ AYA cancer survivors. Disparities in financial burden and mental health can be understood through the four levels of influencing factors in the SGM health disparities research framework: societal, community, interpersonal, and individual (40). Our findings relate to the individual and interpersonal factors such as self-acceptance and the coming out process. Specifically, participants reported not coming out to avoid losing financial support as well as experiencing societal factors such as structural stigma (e.g., employer discrimination and nonaccepting work environments). Our findings can be contextualized further using the conceptualization of stigma as fundamental cause which asserts that stigma, or the co-occurrence of labeling, stereotyping, separation, status loss, and discrimination in the context of power being exercised, is a primary driver of population health disparities (41, 42). Specifically, the mediators or the ways that stigma manifests and leads to disparities (i.e., resources, social isolation, psychological and behavioral responses to stigma, and stress) (41, 42) can be used to further explain our findings as they overlap substantially with the financial hardship domains used to develop our interview guide (29). Suggesting that future research into LGBTQIA+ cancer survivors financial burden should be theoretically driven, incorporating both cancer related financial hardship frameworks and LGBTQIA+ disparities frameworks/theories which may enhance research on the financial impacts of cancer in this population.

LGBTQIA+ AYAs reported significantly worse COST scores than non-LGBTQIA+ AYA survivors. This finding was explained by the interview findings in which participants reported experiencing severe material financial hardship. This is consistent with the well-established literature that a large proportion of cancer survivors experience financial hardship, which is particularly true among AYA survivors who report financial hardship during a dynamic time of development (37). Our findings contribute to the literature in demonstrating that LGBTQIA+ AYAs experienced worse employment outcomes, which has been exacerbated during COVID-19. While financial hardship among LGBTQIA+ survivors has not been explored prior, our findings suggest that LGBTQIA+ AYA survivors may experience different and worse financial hardship than cisgender heterosexual AYAs and older survivors. Our finding regarding stigma and employment discrimination faced by our interview participants is consistent with the literature outside of the cancer context. LGBTQIA+ populations face severe employment discrimination and structural stigma because of their identities throughout the United States (43). Consistent with our findings, and particularly relevant to LGBTQIA+ survivors who are AYA, employees outness impacts the amount of employment discrimination they suffered, with more than one third of LGBTQIA+ individuals reporting not being out at work (43). Further sub-groups of the LGBTQIA+ community are at an elevated risk for employment discrimination including transgender individuals (44). While employment discrimination and stigma surrounding LGBTQIA+ identities vary based on the state and region of the United States, nowhere is free from either (45, 46). Further, educational attainment of LGBTQIA+ and non-LGBTQIA+ AYAs in our sample differed substantially. Education was not a concept that emerged in our qualitative findings but is a known predictor of economic outcomes and differential treatment in the healthcare system in other minority populations and warrants further inquiry (47). Our findings suggest that an LGBTQIA+ identity may substantially worsen the financial hardship experienced by AYA survivors due to the added hurdle of LGBTQIA+ stigma and employment discrimination. As sexual orientation and gender identity data becomes more commonly collected, quantifying the economic impact of LGBTQIA+ AYA disparities is of the upmost importance.

In addition, participants reported behavioral financial hardship including alterations to their saving and spending habits, reliance on caregivers, cost conversations with providers, as well as rationing prescription medications. While behavioral responses to financial hardship have been reported by AYA survivors regardless of sexual orientation or gender identity, some responses may be highly influenced by an LGBTQIA+ identity. Specifically, the reliance on caregivers for financial support is complicated for LGBTQIA+ AYA survivors as families do not always accept LGBTQIA+ identities (48). This familial non-acceptance is represented in our findings regarding an individual who lost familial financial support after coming out resulting in homelessness and medication rationing and another participant who reported hiding their identity for fear of losing familial financial support. Homelessness among LGBTQIA+ youth is not uncommon resulting in an estimated 20-40% of homeless youth identifying as LGBTQIA+ (49, 50). Furthermore, medication rationing due to cost was reported by multiple LGBTQIA+ AYAs in our study. In the literature medication non-adherence is a fairly common behavioral response to financial hardship and has severe and life-threatening consequences (51–53). Future inquiry should explore LGBTQIA+ survivors medication adherence and long-term survival as well as ways to support survivors who face familial non-acceptance. Further our findings suggest that cancer centers should create formal relationships with LGBTQIA+ community organizations in order to more directly support survivors who lose their caregiver support due to their identity. Additionally, further studies are needed to quantify the economic impact of cancer among LGBTQIA+ populations, to support LGBTQIA+ survivors who lose familial support, and to provide robust population specific mental health services to LGBTQIA+ survivors.

In addition to financial burden, LGBTQIA+ AYAs reported significantly higher stress, anxiety, and depression than non-LGBTQIA+ AYA survivors in the survey. These findings were contextualized by participant’s descriptions of their financial stress, which was often described alongside existing mental health challenges, primarily due to prior trauma and other factors involving their LGBTQIA+ identity. Due to identity related conflict with their caregiver, one participant reported suicidality during the interview; most participants reported other significant mental health challenges. The minority stress model suggests that the stressors experienced by LGBTQIA+ populations positions them at an increased risk for mental health issues such as depression, anxiety, and suicidality (54). Our findings suggest that cancer centers should assess survivor mental health and have specific strategies to support LGBTQIA+ AYAs before mental health challenges arise. Stress experienced by participants, heightened by financial burden may differ based on other intersecting identities. For example, one participant did not want to come out due to already feeling marginalized as a person of color. Thus, our findings support the need for a more intersectional approach to financial burden and LGBTQIA+ disparities in cancer research and further exploration into how race, gender, ability, and sexuality all concurrently influence minority stress in the cancer context (55, 56).

### 4.1 Limitations

Our study has limitations including the changing nature of COVID-19 during the data collection periods. Further recall bias may be present as interviews were conducted several months after the survey data were collected. Bias may have been introduced during interview recruitment as individuals who agreed to participate may have fundamentally different experiences than those who did not participate. Our survey lacked the racial and ethnic diversity needed to perform sub-analyses among racial and ethnic minority LGBTQIA+ AYA survivors. Our interview sample size was fairly small; however, it provided the first in-depth exploration of financial burden in LGBTQIA+ AYA survivors and was more racially and ethnically diverse than the survey sample. Overall, the limitations to this study are far outshined by the novel findings.

## 5 Conclusions

This study is the first in-depth exploration of financial burden among LGBTQIA+ AYA cancer survivors. LGBTQIA+ AYA cancer survivors experienced worse financial and mental health outcomes during the COVID-19 pandemic. Financial burden and mental health in our findings were highly complex and intertwined for LGBTQIA+ AYA survivors due to the unique compounding impacts of cancer treatment, COVID-19, and economic instability caused by LGBTQIA+ identity-based stigma. Given their multiple intersecting identities and potential for marginalization, LGBTQIA+ AYA survivors deserve prioritization in research to help reduce financial and psychological distress throughout the cancer continuum.

## Data Availability Statement

The datasets presented in this article are not readily available because informed consent for data sharing was not obtained. Requests to access the datasets should be directed to erin.kent@unc.edu.

## Ethics Statement

The studies involving human participants were reviewed and approved by University of Utah Institutional Review Board (IRB#00091443). The patients/participants provided their informed consent to participate in this study.

## Author Contributions

Conceptualization: ARW, ELW, EEK, and ACK; Methodology: AW, SB, EW, HKK, EEK, and ACK; Software: ACK and ELW; Formal analysis: ARW, SB, and ELW; Writing—original draft preparation: ARW; Writing—review and editing: ARW, ELW, SB, HKK, EEK, and ACK; Supervision: EEK, ELW, and ACK; Project administration: ARW; Funding acquisition: ACK. All authors contributed to the article and approved the submitted version.

## Funding

The research reported in this publication was supported by the Huntsman Cancer Institute Utah Grand Challenges Grant, University of Utah College of Nursing, and the National Cancer Institute of the National Institutes of Health (NIH) under Award Number P30CA042014.

## Author Disclaimer

The content is solely the responsibility of the authors and does not necessarily represent the official views of the Huntsman Cancer Institute, University of Utah, or NIH.

## Conflict of Interest

The authors declare that the research was conducted in the absence of any commercial or financial relationships that could be construed as a potential conflict of interest.

## Publisher’s Note

All claims expressed in this article are solely those of the authors and do not necessarily represent those of their affiliated organizations, or those of the publisher, the editors and the reviewers. Any product that may be evaluated in this article, or claim that may be made by its manufacturer, is not guaranteed or endorsed by the publisher.
